# A Case Study of Psychogenic Non-Epileptic Seizures in a patient with Dependent Personality Disorder

**DOI:** 10.1192/j.eurpsy.2022.1180

**Published:** 2022-09-01

**Authors:** M.F.A. Malik, B. Najeeb, A. Ishaque

**Affiliations:** Rawalpindi Medical University, Institute Of Psychiatry, Rawalpindi, Pakistan

**Keywords:** Case study, Psychogenic Nonepileptic Seizures, Dependent Personality Disorder

## Abstract

**Introduction:**

Psychogenic Nonepileptic Seizures (PNES) refer to the dissociative condition which resembles seizures but does not involve epileptic synchronous cortical activity (Huff, 2021). 20% of people visiting epilepsy clinics have PNES (Huff, 2021). Depression, anxiety, and personality disorders predispose towards PNES (Ekanayake, 2018).

**Objectives:**

To present a case of PNES in a patient with dependent personality disorder (DPD) and to discuss the sociocultural aspects.

**Methods:**

A case study.

**Results:**

A 23-years old, married female presented with 20 days history of episodes of ‘falling down, rolling on ground, and involuntary movements of her head.’ The episodes typically lasted from 20-25 minutes. During the episodes, patient closed her eyes but remained conscious and expressed her distress with gestures, and tearfulness was also observed. Her condition improved when she was offered water. The clinical picture of these episodes evolved with time. Her EEG and serum prolactin levels following the episodes were normal. Accordingly, a diagnosis of PNES was made. No acute stressor was present. The patient also fulfilled the criteria of Dependent Personality Disorder (DPD) (American Psychiatric Association, 2013). During communication with the patient, it appeared that the patient and her attendants perceived the train of questioning as investigational rather than therapeutic. Efforts were made towards a more empathetic understanding of their point of view, and the tailoring of long-term management in accordance with their sociocultural context.

**Conclusions:**

The socio-cultural context is important in the management of PNES and a sensitive, and collaborative approach is recommended. Assessment of personality should be considered in patients presenting with PNES.

**Disclosure:**

No significant relationships.

